# Supplementary material for: The genomic and epigenomic evolutionary history of papillary renal cell carcinomas
Source: Nat Commun. 2020 Jun 18;11:3096. doi: 10.1038/s41467-020-16546-5 (PMC7303129; doi:10.1038/s41467-020-16546-5)
Supplement: Supplementary file 3 — Description of Additional Supplementary Files [file 41467_2020_16546_MOESM3_ESM.pdf]

Description of Additional Supplementary Files

File Name: **Supplementary Data 1**
Description: Coverage information for whole-genome sequencing data. The annotations for each column in tab "WGS_metrics" are included in tab "Note".

File Name: **Supplementary Data 2**
Description: List of sample IDs. IDs for samples in each genomic and epigenomic analysis, including whole-genome sequencing, genome-wide methylation and SNP array genotyping, and deep targeted sequencing.

File Name: **Supplementary Data 3**
Description: Gene list for targeted sequencing (from Lawrence, et al. 2014).

File Name: **Supplementary Data 4**
Description: Non-synonymous single nucleotide variants and related functional annotation. Non-synonymous single nucleotide variants were based on whole genome sequencing and/or deep target sequencing.

File Name: **Supplementary Data 5**
Description: Insertions and deletions (indels) in previously reported cancer driver genes with their functional annotation.

File Name: **Supplementary Data 6**
Description: Potentially deleterious germline variants in cancer susceptibility genes

File Name: **Supplementary Data 7**
Description: Cancer cell fraction (CCF) estimates for each tumor subclone. Estimates are based on the PyClone algorythm. SD: standard deviation.

File Name: **Supplementary Data 8**
Description: Segmentation of copy number alterations based on whole genome sequencing. Segmentation estimates are estimated using the FACETS algorithm. The annotation of columns in tab "Facets_cncf_info" is included in tab "Note".

File Name: **Supplementary Data 9**
Description: Clonality of copy number alterations. For each sample, the proportion of chromosomes carrying clonal and subclonal copy number alterations are shownas estimated by FACETS and Batternberg, respectively.

File Name: **Supplementary Data 10**
Description: Annotation of CDKN2A deletion segment. The locations of the deletion segment are listed for each sample, based on whole genome sequencing and genotyping data.

File Name: **Supplementary Data 11**
Description: The timing of driver mutations relative to somatic copy number gain or loss of heterozygosity (LOH)

File Name: **Supplementary Data 12**
Description: Structural variants (SVs) identified by the Meerkat algorithm. The annotation of columns in tab fusion_list_all" is provided in the Meerkat User Manual (http://gensoft.pasteur.fr/docs/Meerkat/0.185/Manual_0.185.pdf).

File Name: **Supplementary Data 13**
Description: Retrotransposition events identified by TraFiC. The annotation of columns in tab "TE_trafic" is included in tab "Note".

File Name: **Supplementary Data 14**
Description: Number of SNVs contributing to SNV mutational signatures in each sample. Mutational signatures are estimated based on the Sigprofile.

File Name: **Supplementary Data 15**
Description: Telomere length estimates based on whole-genome sequencing data. Telomere length is estimated using the Telseq algorithm. The annotation of columns in tab "Final-telseq" is included in tab "Note".

File Name: **Supplementary Data 16**
Description: Number of indels contributing to indel mutational signatures.
