# Supplementary material for: The genomic and epigenomic evolutionary history of papillary renal cell carcinomas
Source: Nat Commun. 2020 Jun 18;11:3096. doi: 10.1038/s41467-020-16546-5 (PMC7303129; doi:10.1038/s41467-020-16546-5)
Supplement: Supplementary file 20 — Reporting Summary [file 41467_2020_16546_MOESM20_ESM.pdf]

nature research

Corresponding author(s): Maria Teresa Landi

Last updated by author(s): Apr 27, 2020

Reporting Summary

Nature Research wishes to improve the reproducibility of the work that we publish. This form provides structure for consistency and transparency in reporting. For further information on Nature Research policies, see Authors & Referees and the Editorial Policy Checklist .

Statistics

For all statistical analyses, confirm that the following items are present in the figure legend, table legend, main text, or Methods section.

n/a	Confirmed	
☐	☒	The exact sample size (*n*) for each experimental group/condition, given as a discrete number and unit of measurement
☐	☒	A statement on whether measurements were taken from distinct samples or whether the same sample was measured repeatedly
☐	☒	The statistical test(s) used AND whether they are one- or two-sided<br>*Only common tests should be described solely by name; describe more complex techniques in the Methods section.*
☐	☒	A description of all covariates tested
☐	☒	A description of any assumptions or corrections, such as tests of normality and adjustment for multiple comparisons
☐	☒	A full description of the statistical parameters including central tendency (e.g. means) or other basic estimates (e.g. regression coefficient) AND variation (e.g. standard deviation) or associated estimates of uncertainty (e.g. confidence intervals)
☐	☒	For null hypothesis testing, the test statistic (e.g. *F*, *t*, *r*) with confidence intervals, effect sizes, degrees of freedom and *P* value noted<br>*Give P values as exact values whenever suitable.*
☒	☐	For Bayesian analysis, information on the choice of priors and Markov chain Monte Carlo settings
☒	☐	For hierarchical and complex designs, identification of the appropriate level for tests and full reporting of outcomes
☐	☒	Estimates of effect sizes (e.g. Cohen's *d*, Pearson's *r*), indicating how they were calculated

Our web collection on statistics for biologists contains articles on many of the points above.

Software and code

Policy information about availability of computer code

Data collection	No software is used in data collection
Data analysis	Statistical analyses were performed with R version 3.5.1; all statistical tests were two-sided; other software used are listed as follows: Picard tools (v2.1.0), GATK (Genome Analysis Toolkit, v3.6), MuTect (v1.1.7), Bam-readcount (Count DNA sequence reads in BAM files, v0.8.0), oncotator (v1.9.1), gnomAD (The Genome Aggregation Database, v2.1.1), Battenberg (Subclonal copy number estimation, v2.2.8), ASCAT (v2.5.2), FACETS (v0.5.2), PHYLIP (v3.6), Phangorn (Phylogenetic Reconstruction and Analysis, v2.6.0), ape (analysis of phylogenetics and evolution, v5.3), SCHISM (SubClonal Hierarchy Inference from Somatic Mutations, v1.1.2), PyClone (Probabilistic model for inferring clonal population structure from deep NGS sequencing, v0.13.0), DPClust (Dirichlet Process based methods for subclonal reconstruction of tumors, v2.2.8), Svclone (To infer and cluster cancer cell fractions (CCFs) of structural variant (SV) breakpoints, v0.2.2), Palimpsest (studying mutational and structural variant signatures along clonal evolution in cancer, v1.0.0), SigProfiler (v2.5.1.7), Meerkat (v0.185), TraFiC (v1.1.0), RnBeads (Comprehensive analysis of DNA methylation data, v3.9), Minfi (analyze illumnina infinium DNA methylation arrays, v1.30.0)

For manuscripts utilizing custom algorithms or software that are central to the research but not yet described in published literature, software must be made available to editors/reviewers. We strongly encourage code deposition in a community repository (e.g. GitHub). See the Nature Research guidelines for submitting code & software for further information.

Data

Policy information about availability of data

All manuscripts must include a data availability statement. This statement should provide the following information, where applicable:

- Accession codes, unique identifiers, or web links for publicly available datasets
- A list of figures that have associated raw data
- A description of any restrictions on data availability

The whole genome sequencing data, Methylation EPIC data, genotyping data and target-sequencing data have been deposited in the database of Genotypes and

Phenotypes (dbGaP) under accession code phs001573.v1.p1; study website: https://www.ncbi.nlm.nih.gov/projects/gap/cgi-bin/study.cgi?study_id=phs001573.v1.p1

Field-specific reporting

Please select the one below that is the best fit for your research. If you are not sure, read the appropriate sections before making your selection.

- [x] Life sciences
- [ ] Behavioural & social sciences
- [ ] Ecological, evolutionary & environmental sciences

For a reference copy of the document with all sections, see nature.com/documents/nr-reporting-summary-flat.pdf

Life sciences study design

All studies must disclose on these points even when the disclosure is negative.

Sample size	The study population comprise 39 patients with kidney cancers, including 23 with papillary type 1 (pRCC1); 12 with papillary type 2 (pRCC2); and one each with collecting duct tumor (cdRCC); renal fibrosarcoma rSRC; mixed pRCC1/pRCC2 and an unclassified renal cancer with mixed features of pRCC2 and cdRCC (mixRCC). We performed no sample size calculations. We described the genomic landscape of all the tumor samples we could collect of these rare subtypes. Some of these subtypes had never been characterized with genomic data before, and so even a small sample size provides an important contribution to our understanding of kidney cancer.
Data exclusions	Based on DNA sample availability and at least 70% tumor nuclei at histological examination, we excluded some samples and conducted whole genome sequencing (WGS) on 124 samples from 29 subjects, deep targeted sequencing on 139 samples from 38 subjects, SNP array genotyping on 101 samples from 38 subjects, and genome-wide methylation profiling on 139 samples from 28 subjects. Exclusion criteria were based on low percentage of tumor nuclei, high percentage of necrosis, insufficient high quality DNA for some of the assays. The criteria were pre-established.
Replication	We validated four in-frame fusions by reverse transcription and PCR-based sequencing, and 303 additional structural variants by Ion Torrent PGM Sequencing using a custom AmpliSeq primer pool. Subsets of SNVs detected by WGS data were validated by targeted sequencing. RT-PCR was repeated twice for reproducibility from the same cDNA library.
Randomization	N/A. There is no comparison between a treatment and a placebo.
Blinding	N/A. This is not a clinical trial with no treatment allocation.

Reporting for specific materials, systems and methods

We require information from authors about some types of materials, experimental systems and methods used in many studies. Here, indicate whether each material, system or method listed is relevant to your study. If you are not sure if a list item applies to your research, read the appropriate section before selecting a response.

Materials & experimental systems

n/a	Involved in the study	
x		Antibodies
x		Eukaryotic cell lines
x		Palaeontology
x		Animals and other organisms
	x	Human research participants
x		Clinical data

Methods

n/a	Involved in the study	
x		ChIP-seq
x		Flow cytometry
x		MRI-based neuroimaging

Human research participants

Policy information about studies involving human research participants

Population characteristics	See above about the subtypes of kidney cancers. All tumors were treatment-naive. We used a study design with multiple tumor samples taken at a distance of ~1.5 cm from each other starting from the center of the tumor towards the periphery, plus multiple samples from the most proximal to most distant area outside the tumor. When present, we also collected multiple samples from metastatic regions outside the kidney (adrenal gland). We have described the following covariates in Supplementary Table 1: age at diagnosis, sex, tumor size, tumor stage, and follow-up time
Recruitment	This study was based on archived samples collected at the Regina Elena Cancer Institute, Rome, Italy. There was no sample selection; these were the samples from the only patients who were examined at the clinic and had these rare kidney cancer subtypes
Ethics oversight	Written informed consent to allow banking of biospecimens for future scientific research was obtained from each subject. This work was excluded from the NCI IRB Review per 45 CFR 46 and NIH policy for the use of specimens/data by the Office of Human Subjects Research Protections (OHSRP) of the National Institutes of Health. The data was anonymized.

Note that full information on the approval of the study protocol must also be provided in the manuscript.
